# Case Report: A period-based upper limb rehabilitation program using a degrees-of-freedom constraint strategy in severe post-stroke hemiparesis

**DOI:** 10.3389/fresc.2026.1785433

**Published:** 2026-03-23

**Authors:** Norikazu Hishikawa, Koshiro Sawada, Yuki Iwasaki, Satoru Kakoyama, Yu Iitsuka, Yoshiaki Komatsu, Hiroshi Maeda, Yasuo Mikami

**Affiliations:** 1 Department of Rehabilitation Medicine, Graduate School of Medical Science, Kyoto Prefectural University of Medicine, Kyoto, Japan; 2 Department of Rehabilitation, Gakusai Hospital, Kyoto Interdisciplinary Institute of Community Medicine, Kyoto, Japan; 3 Department of Clinical Services, Rakuhoku Prosthetic Orthotic Manufacturing Co., Ltd., Kyoto, Japan; 4 Life Step Service Co., Ltd., Saitama, Japan

**Keywords:** case report, degrees of freedom, motor learning, severe hemiparesis, stroke, upper limb

## Abstract

**Background:**

Severe upper limb hemiparesis after stroke is often characterized by impaired motor function, increased flexor tone, and abnormal motor coordination, resulting in limited functional reaching. Because reaching requires coordinated control of joints, conventional task-oriented training may not sufficiently address motor control deficits arising from excessive or poorly regulated joint degrees of freedom (DoF). This case report describes a period-based upper limb rehabilitation program incorporating a constraint strategy targeting DoF to facilitate motor recovery in a patient with severe post-stroke hemiparesis.

**Case description:**

A 50-year-old man with left upper limb hemiparesis secondary to right putaminal hemorrhage (163 days post-onset) presented with severe impairment (Fugl–Meyer Assessment for Upper Extremity motor score, 12 points) and spasticity (Modified Ashworth Scale 2–3 in shoulder internal rotators, elbow flexors, and wrist flexors). Insufficient selective motor control and increased spasticity resulted in a dominant upper limb flexion synergy pattern, limiting his ability to perform forward reaching.

**Therapeutic intervention:**

A structured, period-based program was implemented over 21 consecutive days (60 min/day) with a proximal-to-distal progression and progressive release of movement constraints from the shoulder to the elbow and then to the wrist and fingers. Gravity-load management and DoF constraints were provided using an arm support device and a wrist–hand–finger orthosis in the early periods. As proximal voluntary control emerged, the wrist–hand–finger orthosis was replaced by a dynamic finger extension orthosis. In addition, neuromuscular electrical stimulation was applied to facilitate selective muscle activation across training periods.

**Follow-up and outcomes:**

Spasticity of the paretic upper limb decreased progressively over the training period, with early reductions in proximal muscle tone followed by later reductions in distal spasticity. Improvements in passive joint range of motion and consistent reductions in joint pain were observed throughout the intervention. Subsequently, motor function improved, as reflected by an increase in the Fugl–Meyer motor score to 16 points, with reduced synergistic movement patterns and more controlled reaching during tasks.

**Conclusion:**

An upper limb rehabilitation framework incorporating a DoF constraint strategy may support the recovery of coordinated motor control through a structured, period-based approach in individuals with severe post-stroke hemiparesis.

## 1 Introduction

Post-stroke upper limb paresis limits activities of daily living, such as eating, grooming, and dressing, and consequently reduces quality of life (1). This impairment is primarily characterized by voluntary movement disorders resulting from corticospinal tract damage. Additionally, impaired coordination, sensory disturbance, and spasticity collectively contribute to functional decline in the paretic upper limb.

Approximately 70% of stroke survivors present with upper limb paresis in the early phase after onset, and only approximately 12% regain functional hand use after 6 months, indicating a poor prognosis for recovery (2, 3). In addition to corticospinal tract injury, interhemispheric inhibition due to imbalanced inhibitory interactions between the cerebral hemispheres, as well as learned non-use—a phenomenon in which repeated failure and decreased use of the paretic limb leads to underutilization of residual function—also contribute to persistent motor impairment (4–6). Therefore, early intervention is crucial to prevent the development of learned non-use and to promote the use of residual motor function. Moreover, recovery of upper limb function remains a highly valued rehabilitation goal and often becomes increasingly important as limitations in daily activities persist over time after stroke (7).

In motor learning theory, the principles of task specificity, optimal task difficulty, and appropriate constraint of the degrees of freedom (DoF) are fundamental to facilitating skill acquisition and functional recovery (8). Task-specific, repetitive training reinforces movement patterns relevant to daily activities and promotes neural reorganization, whereas an optimal level of task difficulty is essential for inducing neuroplastic changes. Neuroplasticity is promoted by intensive, task-specific motor training and contributes to functional recovery after cortical injury through use-dependent reorganization of movement representations in the primary motor cortex (9, 10). However, when voluntary movement is impaired, excessive motor complexity may increase task difficulty beyond an optimal level, thereby limiting the effectiveness of task-specific training (11). In this context, temporarily constraining the DoF during movement tasks simplifies the motor control problem, particularly in individuals with impaired voluntary movement. In severe hemiparesis, attempts at multijoint movement frequently elicit abnormal flexion synergies and excessive co-contraction, partly because of corticospinal tract dysfunction. Under such conditions, unrestricted release of multiple DoF may increase motor variability and reinforce maladaptive movement patterns. Therefore, structured regulation of DoF may reduce motor complexity, limit the expression of abnormal synergy patterns, and provide a more stable foundation for the gradual re-emergence of selective motor control. This DoF constraint strategy enables learners to focus on controlling a limited number of joints or movement components, thereby enhancing movement accuracy and stability during the early stages of skill acquisition (12). In patients with severe upper limb paresis, voluntary movement of the paretic limb is often minimal or absent, making the reacquisition of the sensation that the paretic limb “can move” a crucial first step in motor learning. The DoF constraint strategy may help evoke this experience of controllable movement by minimizing excessive effort and promoting successful movement experiences. However, despite the widespread use of lower-limb orthoses and robotic-assisted devices in gait training, where joint motion is externally guided or mechanically constrained to enable repetitive practice (13, 14), its clinical application in upper limb rehabilitation after stroke has rarely been reported.

This report describes a middle-aged man with severe upper limb hemiparesis following putaminal hemorrhage, characterized by marked spasticity, abnormal flexion synergy, and severely limited voluntary reaching, who underwent a period-based upper limb rehabilitation program incorporating a DoF constraint strategy.

## 2 Case description

A 50-year-old right-handed man presented to a local clinic with headache and was subsequently transported by ambulance to an acute care hospital, where head computed tomography revealed a right putaminal intracerebral hemorrhage. During the acute phase, craniotomy, hematoma evacuation, and additional decompressive surgery were performed. He was transferred to the convalescent rehabilitation ward of our hospital on day 22 after stroke onset, where he received intensive rehabilitation treatment (physical therapy, occupational therapy, and speech-language therapy) for approximately 3 h per day. Baseline assessment (T0) for this case report was conducted 163 days after stroke onset, corresponding to the late subacute stage of recovery (15).

The patient had a history of hypertension and dyslipidemia, both of which were well controlled on appropriate medications. His cognitive function was intact, as indicated by a Mini-Mental State Examination score of 30. Activities of daily living were largely independent, with a Functional Independence Measure motor score of 74. Left upper limb motor function was assessed using the Stroke Impairment Assessment Set, with a score of 2 on the Knee-Mouth test and 1a on the Finger Function test, and was characterized by voluntary shoulder elevation limited to the nipple level and finger motion dominated by an involuntary mass flexion pattern without isolated motor control. In addition, spasticity of the affected upper limb was pronounced, with a Modified Ashworth Scale (MAS) score of 2–3 in the shoulder internal rotators, elbow flexors, and wrist flexors. The patient hoped to be able to perform a forward reach with his left hand to support a book while reading. However, insufficient selective motor control and increased spasticity resulted in a dominant upper limb flexion synergy pattern, which limited his ability to reach forward.

## 3 Therapeutic rehabilitation intervention

**Figure 1:**
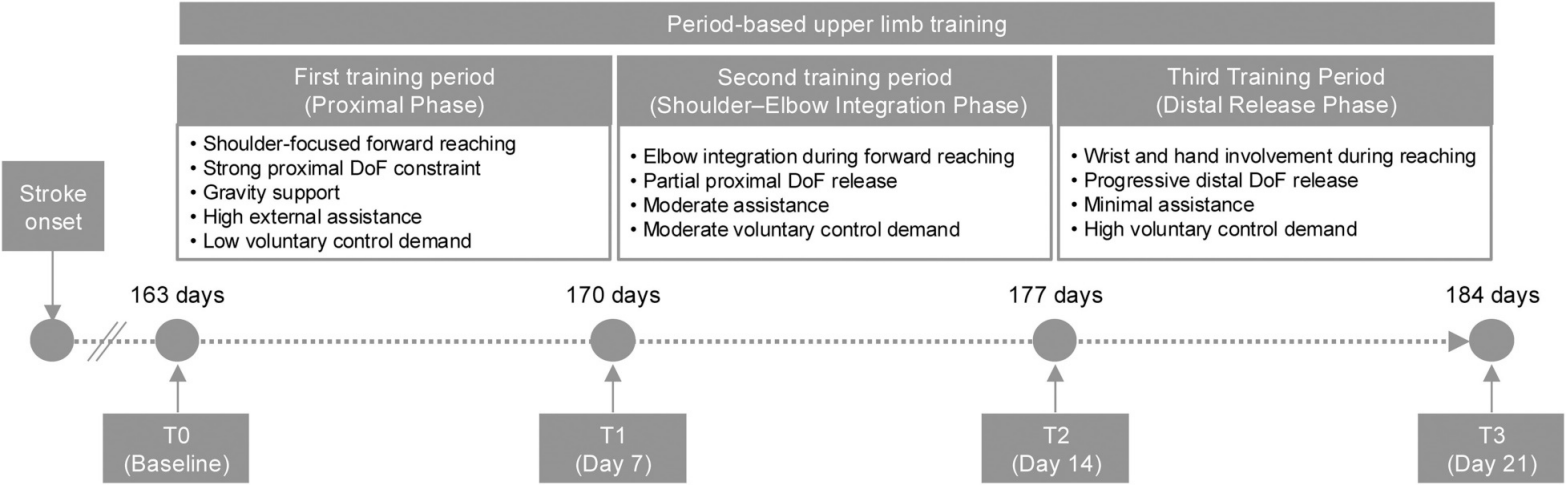
Timeline and training structure of the period-based upper limb rehabilitation program. The patient was admitted to a convalescent rehabilitation ward 22 days after stroke onset. A period-based upper limb rehabilitation program was initiated 163 days after onset and consisted of three consecutive 7-day training periods with progressive proximal-to-distal release of joint DoF. Clinical assessments were conducted at baseline (T0) and after each training period (T1–T3). DoF, degrees of freedom.

During hospitalization, a period-based upper limb rehabilitation program consisting of three stages (the first, second, and third training periods) was implemented as part of occupational therapy. Each training period consisted of daily 60-minute sessions for 7 days, and all periods were designed to improve forward-reaching movements. The patient was assessed at four time points: T0 (baseline), and 7 days (T1), 14 days (T2), and 21 days (T3) after initiation of the rehabilitation program (corresponding to 170, 177, and 184 days after stroke onset, respectively). An additional follow-up assessment was conducted at discharge to home, 27 days after T3 (211 days after stroke onset). Clinical outcomes were evaluated using the Fugl–Meyer Assessment for Upper Extremity (FMA-UE), which assesses motor and sensory function, passive joint motion, and joint pain, and the MAS. A case report timeline is shown in Figure 1.

**Figure 2:**
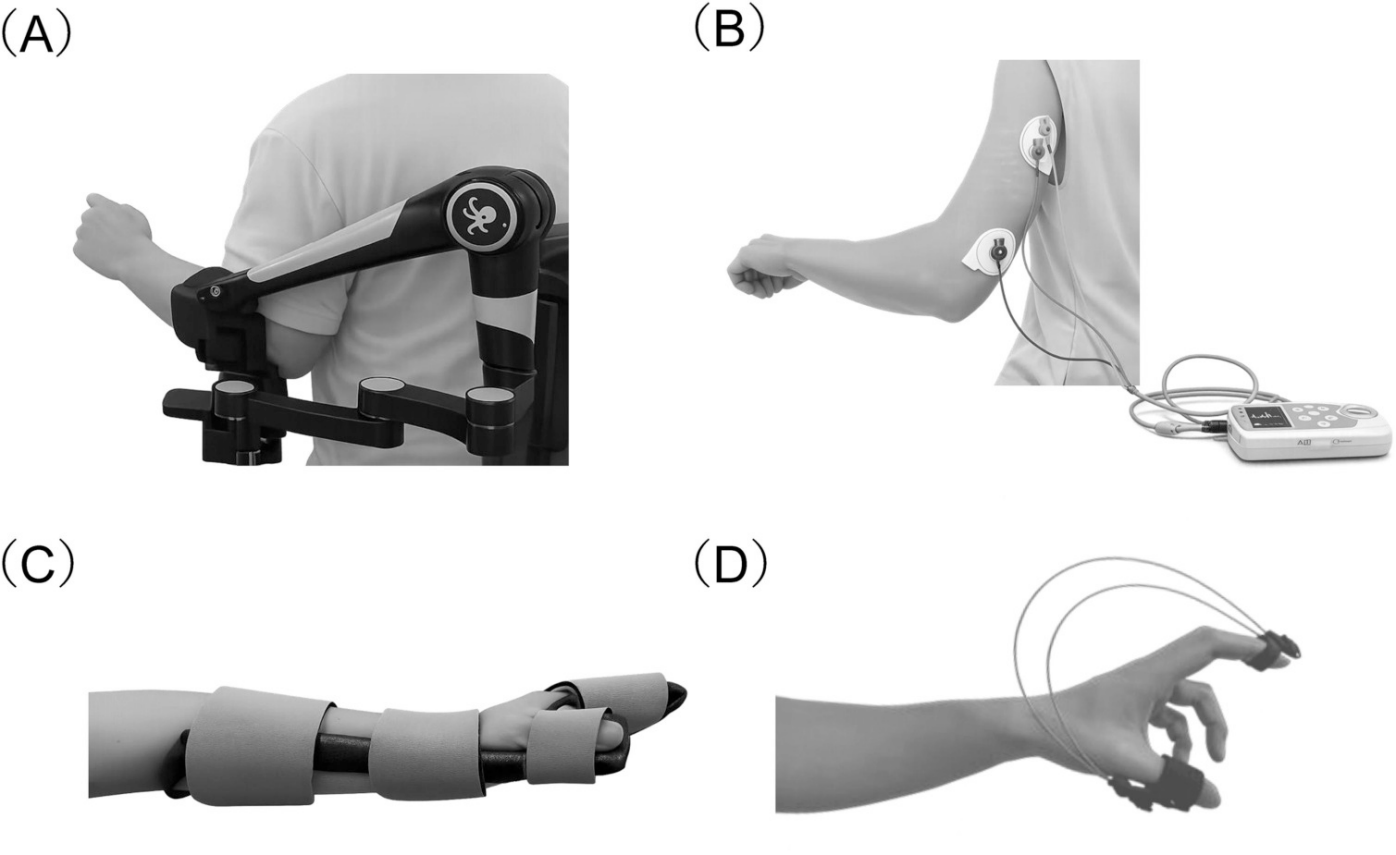
Structural and functional details of the devices used in the upper limb rehabilitation program. **(A)** Arm support device (ORTHOPUS Supporter; ORTHOPUS SAS, Nantes, France) used to reduce gravitational load during reaching tasks. **(B)** EMG-triggered neuromuscular electrical stimulation device (IVES; OG Wellness Technologies Co., Ltd., Okayama, Japan), which detects voluntary EMG activity and delivers electrical stimulation proportional to muscle activation to facilitate voluntary contraction. **(C)** WHFO providing adjustable wrist stabilization. **(D)** DFEO used to assist finger extension. These devices were applied in combination according to the training phase described in Figure 1 to systematically regulate joint DoF and movement complexity. EMG, electromyography; IVES, integrated volitional-control electrical stimulator; WHFO, wrist–hand–finger orthosis; DFEO, dynamic finger extension orthosis; DoF, degrees of freedom.

As shown in Figure 2, four assistive devices were used during the training program for the purpose of assisting, facilitating, and stabilizing the paretic limb: an arm support device, a neuromuscular electrical stimulation device, a wrist–hand–finger orthosis (WHFO), and a dynamic finger extension orthosis (DFEO). An arm support device (ORTHOPUS Supporter; ORTHOPUS SAS, Nantes, France) was used to reduce the antigravity load of the affected upper limb and facilitate forward-reaching movements. This robotic upper limb assistive device provides adjustable gravity compensation and offers two operating modes: a free mode, which enables dynamic arm movement with minimal resistance, and stationary mode, which allows the arm to be positioned and maintained at a desired height during task-specific training. Unlike conventional spring balancer systems, which may provide variable support across the movement trajectory, the arm support device used in this program enabled consistent gravity compensation while maintaining stable support during voluntary reaching movements (16). Additionally, the Integrated Volitional-control Electrical Stimulator (IVES; model GD-611, OG Wellness Technologies Co., Ltd., Okayama, Japan), a neuromuscular electrical stimulation device with closed-loop electromyographic (EMG) control was used. This device enables both stimulation and EMG recording through the same electrode array, delivering electrical stimulation proportional to voluntary EMG activity to assist voluntary muscle contraction (17). Furthermore, depending on the training phase, either a custom-made static WHFO fabricated from casting material (fiberglass casting tape coated with water-curable polyurethane resin) or a DFEO was used. The static WHFO was intended to maintain the wrist and/or fingers in a functional position and to inhibit flexor spasticity patterns commonly observed in patients with stroke (18). In contrast, the DFEO was intended to provide gentle extension assistance via elastic finger loops, allowing voluntary finger movement while aiming to reduce flexor overactivity (19).

In the first training period, interventions were implemented to facilitate selective shoulder movements on the paretic side based on a motor learning approach using a DoF constraint strategy. The ORTHOPUS Supporter was used to provide gravity compensation, and the IVES was applied to the paretic infraspinatus to enhance scapular stability. A custom-made static WHFO was used to stabilize the wrist and fingers. In the second training period, as voluntary shoulder movement emerged during the task, the IVES was applied to the triceps brachii instead of the infraspinatus to promote elbow extension. Use of the ORTHOPUS Supporter for gravity compensation and the static WHFO for wrist and finger stabilization was continued. During the third training period, as voluntary elbow extension emerged, the IVES was applied to the extensor digitorum instead of the triceps brachii to facilitate wrist extension. During this period, the static WHFO was discontinued and replaced with DFEO. Through these interventions, selective and task-specific training with optimally adjusted difficulty was consistently provided, and controlled reaching movements within a constrained workspace were facilitated. This phase-based progression allowed the gradual release of movement constraints as voluntary control improved. In addition, patient-reported outcome measures of upper limb movement were collected daily during the training sessions. Perceived ease of movement, perceived control of effort, sense of use of the upper limb, and satisfaction with movement were assessed using a numerical rating scale ranging from 0 to 10, where higher scores were defined as indicating greater ease of movement, better effort control, stronger sense of use, and higher satisfaction. These patient-reported measures were included to capture the patient's perceived controllability and usability of the paretic upper limb during task training.

## 4 Follow-up and outcomes

The 21-day period-based training program, comprising three consecutive 7-day training periods, was completed with full adherence (100%) and without any adverse events. Table 1 presents the changes in the clinical outcomes and the patient-reported outcomes across the three training periods.

**Table 1:** Changes in upper limb-related clinical outcomes across each training period.

Outcome measures	T0 (Baseline)	T1 (Day 7)	T2 (Day 14)	T3 (Day 21)	Discharge (Day 48)
FMA-UE score					
Motor function					
Upper limb	12	12	12	12	13
Wrist	0	0	0	0	0
Hand	0	0	3	4	4
Coordination	0	0	0	0	0
Total	12	12	15	16	17
Sensation	6	6	6	6	6
Passive joint motion	15	19	21	21	21
Joint pain	17	21	23	24	24
Spasticity					
MAS score					
Shoulder adductors	3	2	1+	1+	1+
Shoulder internal rotators	3	1+	1+	1+	1+
Elbow flexors	3	2	2	2	2
Forearm supinators	3	2	2	1+	1+
Wrist flexors	3	2	2	1+	1+
Finger flexors	3	2	2	1+	1+

T1–T3 indicate Days 7, 14, and 21 after program initiation (corresponding to 170, 177, and 184 days after stroke onset, respectively). Follow-up assessment was performed at discharge to home (27 days after T3; 211 days after stroke onset). FMA-UE, Fugl–Meyer Assessment for Upper Extremity; MAS, Modified Ashworth Scale.

Clinical outcomes demonstrated gradual improvements over the course of the intervention. At baseline (T0), the patient exhibited severely limited voluntary movement of the paretic upper limb, with a total FMA-UE motor score of 12. At T1, the FMA-UE motor score remained unchanged; however, spasticity of the paretic upper limb, as assessed using the MAS, was markedly reduced overall. Passive joint motion and joint pain also improved at this time point. At T2, the hand subscore increased from 0 to 3, and the total FMA-UE motor score improved to 15. Spasticity in the shoulder adductors further decreased (from 2 to 1+). At T3, the hand subscore increased to 4 and the total FMA-UE motor score improved to 16. In addition, spasticity of the paretic upper limb further decreased, with reductions observed in the forearm supinators and wrist and finger flexors (from 2 to 1+). Sensory function remained unchanged throughout the intervention, whereas passive joint motion and joint pain scores continued to improve over time. In the follow-up assessment conducted at discharge to home (27 days after T3), the total FMA-UE motor score improved to 17, indicating maintenance and slight progression of motor recovery beyond the structured intervention period. Patient-reported assessments showed that perceived ease of movement, perceived control of effort, sense of use of the upper limb, and satisfaction with movement remained consistently high throughout the program, with scores ranging from 9 to 10 on the numerical rating scale.

## 5 Discussion

This case report suggests that a period-based upper limb rehabilitation program incorporating motor learning principles—such as task specificity, optimal task difficulty, and regulation of DoF—may be associated with gradual improvements in motor function, spasticity, and joint pain in the paretic upper limb of a patient with severe post-stroke hemiparesis. These changes occurred despite minimal voluntary movement in the early phase, suggesting that selective motor control may re-emerge even in the severely impaired paretic upper limb when movement complexity is appropriately regulated. Furthermore, the continued functional gains observed during the late subacute stage suggest that improvements in spasticity, joint pain, and joint mobility may reduce biomechanical and sensorimotor constraints on movement, thereby enabling subsequent improvements in motor performance even beyond the period of rapid spontaneous recovery.

From a motor learning perspective, the regulation of excessive DoF is considered a foundational principle for acquiring coordinated movement (12). In the present program, movement complexity was deliberately reduced through external constraints and graded assistance, and constraints were progressively released in a proximal-to-distal sequence (shoulder → elbow → wrist) in accordance with the emergence of voluntary control. This hierarchical progression aligns with established motor learning frameworks that emphasize task simplification, graded task difficulty, and staged skill acquisition (8, 20). Importantly, the intervention did not merely restrict movement but was designed to provide the patient with controllable and successful movement experiences, in which voluntary movement of the paretic limb was perceptible. Such experiences are known to enhance self-efficacy, motivation, and engagement, all of which are critical facilitators of motor learning and neural plasticity (21, 22). In this context, constrained DoF therapy was not intended to restrict movement permanently, but to create a simplified motor environment in which successful, controllable movements could be experienced, thereby supporting motor relearning in the presence of impaired corticospinal control.

Despite the theoretical relevance of DoF constraint strategies, their clinical application in post-stroke upper limb rehabilitation has remained limited. One possible explanation is that, unlike gait training, upper limb movements require fine multijoint coordination across a wide workspace, and excessive constraint may compromise task variability and functional relevance. Moreover, much of the existing evidence in upper limb rehabilitation has focused on interventions targeting distal segments such as the wrist and hand, whereas empirical evidence supporting training strategies that explicitly address multijoint coordination and DoF control of the entire upper limb remains limited (23, 24). Additionally, in severely impaired upper limbs, marked spasticity and abnormal synergies are known to constrain multijoint coordination and voluntary motor control (25, 26). When movement is performed under such insufficient control with an excessive release of DoF, the risk of promoting abnormal movement patterns may increase. The present case suggests that a period-based approach, in which constraints are systematically adjusted in response to emerging voluntary control, may help address these challenges and facilitate the practical and clinically feasible application of DoF constraint strategies in upper limb rehabilitation.

The temporal pattern of recovery further supports the relevance of this approach. During the early-to-middle phases of training, the FMA-UE motor score did not increase; however, a marked reduction in spasticity of proximal muscles was observed. Subsequently, during the mid-to-late phases of training, a substantial reduction in distal muscle spasticity was observed. However, while a slight improvement was observed in the hand subscore, wrist motor activity did not show improvement despite the reduction in spasticity. In this case, reductions in muscle tone did not directly correspond to recovery of selective voluntary wrist control. Although decreased spasticity can reduce passive resistance and abnormal co-contraction, restoration of independent wrist movement requires sufficient corticospinal drive. In severe hemiparesis, distal motor recovery—particularly at the wrist—is highly dependent on corticospinal pathway integrity and may remain limited despite improvements in muscle tone. In addition, throughout the training period, passive joint range of motion progressively improved, and joint pain was consistently reduced. These improvements may be attributable to reductions in excessive muscle activation and abnormal synergies achieved through DoF constraint and graded assistance, which likely decreased mechanical stress and nociceptive input during repeated movement training. It is also possible that, prior to the intervention, repeated attempts at poorly controlled or synergistic movement patterns contributed to excessive muscle activation and joint stress, thereby contributing to elevated muscle tone and pain. By regulating movement complexity, the present approach may have interrupted this maladaptive cycle. In contrast, recovery of voluntary motor function likely depends on neurophysiological reorganization and motor relearning processes, which may require longer training duration and therefore progress more gradually than reductions in muscle tone and joint pain. Taken together, these findings suggest that modulation of muscle tone and pain may reduce both biomechanical and neurophysiological barriers to voluntary movement, thereby creating more favorable conditions for subsequent improvements in selective motor control. In severely impaired upper limbs, where movement attempts often elicit abnormal synergies and co-contraction, the combination of DoF constraint and graded assistance may help maintain movement within a controllable workspace, increasing the likelihood of accurate repetition and facilitating the acquisition of more adaptive movement patterns.

The training goal in this case was not abstract motor recovery but a patient-identified functional activity: reaching forward with the paretic hand to support a book during reading. Improvements in motor function, reductions in spasticity, and sustained high levels of perceived movement controllability likely contributed synergistically to the feasibility of this task. This finding underscores the importance of integrating patient-specific functional goals with structured motor learning principles, particularly in individuals with severe hemiparesis, for whom conventional impairment-based gains may not readily translate into meaningful activity.

A key strength of this case report lies in the transferability of the program design. Although specific devices were employed, the core components—gravity-load management to enable active movement; period-based DoF constraint with planned joint release; high-frequency, task-specific repetition; and continuous monitoring of patient-reported movement controllability—can be implemented using alternative resources such as arm supports, positioning strategies, orthoses, or therapist-assisted guidance. Accordingly, this report offers a conceptual framework for structuring upper limb rehabilitation program in patients with severe hemiparesis, rather than a device-dependent protocol. This conceptual orientation also distinguishes the present approach from conventional upper limb rehabilitation strategies. This case report differs from conventional upper limb rehabilitation approaches in that it explicitly operationalized the regulation of DoF as a central therapeutic principle. Unlike constraint-induced movement therapy (23), which primarily restricts the non-paretic limb to promote use of the affected side, the present program systematically constrained and progressively released joint DoF within the paretic limb itself. Furthermore, while robotic or gravity-support devices are often used to assist movement, they are not typically integrated into a structured, period-based framework grounded in motor learning theory. The novelty of this approach lies not in the individual devices employed, but in the intentional, theory-driven sequencing of movement constraint and release to facilitate the re-emergence of selective motor control in patients with severe hemiparesis. However, several limitations must be acknowledged. This report describes a single case without a control condition, precluding causal inference regarding individual intervention components. Multiple modalities were applied concurrently, making it difficult to isolate their specific effects. Although conventional rehabilitation treatment was initiated in the postoperative period, the period-based DoF constraint upper limb rehabilitation program described in this report began in the late subacute stage. Nevertheless, spontaneous neurological recovery during this phase cannot be entirely excluded as a contributing factor to the observed improvements. Objective electrophysiological assessments, such as surface EMG recordings at predefined assessment time points, were not performed; therefore, changes in muscle activation patterns or neuromuscular control could not be objectively quantified. In addition, patient-reported measures were collected during training sessions and may have been influenced by contextual or motivational factors. Although follow-up at discharge was available, long-term outcomes beyond the post-discharge period were not assessed.

## 6 Conclusion

To accelerate recovery in patients with severe post-stroke upper limb hemiparesis, the potential of an upper limb rehabilitation program incorporating motor learning theory, including a DoF constraint strategy, should be further explored.

## Data Availability

The raw data supporting the conclusions of this article will be made available by the authors, without undue reservation.
